# Near-Newtonian Blood Behavior – Is It Good to Be a Camel?

**DOI:** 10.3389/fphys.2019.00906

**Published:** 2019-07-17

**Authors:** Ursula Windberger, Roland Auer, Monika Seltenhammer, Georg Mach, Julian A. Skidmore

**Affiliations:** ^1^Center for Biomedical Research, Medical University of Vienna, Vienna, Austria; ^2^Center for Forensic Medicine, Medical University of Vienna, Vienna, Austria; ^3^Camel Reproduction Center, Dubai, United Arab Emirates

**Keywords:** camel, horse, exercise, blood fluidity, erythrocytes, shear thickening, viscosity, rheology

## Abstract

From a certain level of exercise-intensity onward, hematocrit increases in horses, which brings more oxygen carriers into the bloodstream. Camels, however, when used in competitive racing could be even in need of iron supplementation and blood transfusions due to a severe reduction of their available hematocrit compared to their resting hematocrit. Since the extrinsic and intrinsic mechanical properties of camel erythrocytes (RBC) are so different compared to RBCs of other mammals, the question arises whether this observation might be a response to endurance exercise aiming at keeping the RBC count low. Rheometry indicated dromedary camel blood to behave almost Newtonian, which is unique amongst mammals. Shear thinning did increase with the hematocrit, but remained marginal compared to horses. As a result, camel whole blood viscosity (WBV) exceeded horse WBV at high shear rates, an effect, which was significantly augmented when the packed cell volume (PCV) was increased. Therefore, in camels any infusion of RBCs into the bloodstream can increase the cardiac work and the energy input into the endothelium more effectively, which should generate vascular remodeling in the long term. Yielding, however, was completely absent in camel blood, confirming low cohesion between its components at quasi-static flow. Camel blood remained a viscous liquid without a threshold even at unphysiologically high PCVs. This can help to washout lactate when camels start to dehydrate and might contribute to the sustained working ability of these animals. The subtle pseudoplastic behavior and the high viscosity contrast across the RBC membrane point to weak coupling between blood flow and red cell behavior. We predict that RBCs flow as separate entities and can show various types of motion, which can lead to friction instead of being collectively aligned to the flow direction. In comparison to horses, this behavior will become relevant at higher RBC counts in front of flow obstacles and possibly cause vascular remodeling if the PCV rises during strenuous exercise, a matter that should be avoided.

## Introduction

For centuries, camels and horses have been used for comparable purposes. They transport loads, their dairy products and meat are food sources in several countries (Abrhaley and Leta, 2018; Ugwu et al., 2019), and they are used for riding and competitive racing. When performing exercise, cardiovascular regulatory mechanisms attempt to meet the rising oxygen demand of the muscle cells by an increase of blood flow, both, systemically through enhanced cardiac output, and locally through the metabolic and mechanical responses of the vascular wall to adjust the vessel diameter. Although the aerobic threshold in the striated muscle cell during exercise depends on a number of cellular factors like fiber type, mitochondrial quantity, and intracellular buffer systems, the oxygen supply via the local blood flow still limits the achievable aerobic capacity (VO_2max_). Surface area of intramuscular microvessels and the flux of RBCs in that geometry play a significant role for blood gas exchange. By the same mechanism, the muscle hemodynamics also influence the extent of lactate accumulation (Hinchcliff et al., 2008) and the fatigue profile. Phases of high-intensity output training generate reactive oxygen species in muscle cells, which aggravates fatigue (Bogdanis, 2012). But exercise also increases muscular heat, and considering the manifold mechanisms of dromedary camels to avoid heat accumulation when they are water deprived (Gaughan, 2011), animals ranging in the desert do not perform high-intensity exercise – except male individuals in rut. Since the dromedary camel has lost its importance as “beasts of burden” long ago, new challenges developed out of, which are camel racing. Nowadays, dromedary camels bred to gain maximum racing performance receive optimal support by their food and training, and can even perform like horses. When horses exercise, their hematocrit increases, which identifies them as “natural blood dopers” (Boening et al., 2011). Training of horses raises also their resting RBC count (Dunstan et al., 2019), circumstances that bring more oxygen carriers into the bloodstream (Persson, 1983; Stoiber et al., 2005). But camels typically do not respond by an increase of the blood oxygen store (Auer et al., 2015), instead, they can even be in need of circulating RBCs (Tinson, 2017), especially when they are infected by blood parasites (Al-Hakak, 2017). Since the hematocrit has a major impact on blood viscosity, blood fluidity changes in horses during a race, but does not in camels. This may be explained by the mechanical RBC properties of horses and camels, which could not be more different.

Equine RBCs present as round discocytes with biconcave lateral faces, typical for the mammalian class, with a volume around 50 fL. The membrane bilayer is high in saturated fatty acids (Plasenzotti et al., 2007), which increases the cohesion between the phospholipids and reduces membrane fluidity. It appears from the electrophoretic behavior that horse RBC membranes are deficient in band 4.2 and band 6 proteins (Baskurt et al., 1997; Guerra-Shinohara and de Baretto, 1999; Matei et al., 2000). Band 6 is a 36 kDa protein (glyceraldehyde-3-phosphate dehydrogenase, G3PD). In human RBCs it is bound to the N-terminal cytoplasmic tail of the major anion exchanger (band 3 protein, AE1) (Rogalski et al., 1989), and therefore it appears in RBC ghosts. It dissociates from band 3 by addition of heme (Salé et al., 2005), high salt concentration (Maretzki et al., 1989), and certain metabolites (Tsai et al., 1982), to become the active form. The question, if the electrostatic association between band 3 and band 6 affects membrane deformability, is still unresolved as yet, but it has been reported that G3PD maintains membrane stability at low pH (Gedde et al., 1999). Pure and purified G3PD from different sources interferes with microtubule bundling and actin polymerization (Sirover, 1999), therefore it cannot be excluded that the enzyme has an impact on the mechanical behavior of RBCs, at least in non-mammalian RBCs that contain a membrane-associated tubulin system. Protein 4.2 is a 72 kDa polypeptide (Gardner and Bennett, 1989) adhered to the cytoplasmic domain of band 3 proteins that also interacts with ankyrin and CD47, the latter being a protein of the Rh-complex. By linking AE1 to the Rh-complex, protein 4.2 stabilizes the cell membrane (Lux, 2016). Its absence due to natural mutations or in protein 4.2-gene knockout mice causes hereditary spherocytosis (Bruce et al., 2002; Dahl et al., 2004; Satchwell et al., 2009).

In contradiction though, horse RBCs are susceptible to the formation of echinocytes. The underlying reason is still poorly investigated or understood, as the RBC membrane composition (both proteins and lipids) does not explain this characteristic. Other candidates that can promote echinocytes formation are imbalances in the magnitude of the leaflets (Nakao, 2002), incomplete membrane shedding that hinders the formation of spherocytes, or hemoglobin-associated factors like those that generate poikilocytosis in goats (Lorkin, 1973). Possibly the answer does not lie in compositional issues, but in RBC function. Generally, echinocyte formation is a natural process during aging and paralleled by a loss of intracellular ATP. Low ATP leads to increased membrane tension by strengthening the spectrin-membrane connection, which can result in membrane blebs (Sens and Gov, 2007; Betz et al., 2009). Clinical studies showed that equine echinocytosis occurs at several rather unrelated circumstances like electrolyte depletion (Geor et al., 1993; Weiss and Geor, 1993), colitis and local infections (Weiss and Geor, 1993; Weiss and Moritz, 2003), during competitive races, and at furosemide treatment (Fedde and Wood, 1993; Weiss et al., 1994). While it is not unexpected that the contact of RBCs with endotoxic lipopolysaccharide promotes the formation of echinocytes (Warren et al., 1983), it is astonishing that exercise favors the generation of echinocytes. Echinocytosis can even persist for several hours after a race. The question arises whether this causes any patho-physiological consequences, or if it simply represents RBC damage. Recent studies on transgenic mice have shown that ATP discharges from RBCs when the hemoglobin oxygenation decreases. The effect is mediated via deoxy-hemoglobin binding to band 3 protein (Chu et al., 2016). Subsequently, ATP in plasma binds to the P2Y receptor of endothelial cells and thereby introduces NO synthesis, which in turn causes vasodilation. Although the price to pay is reduced deformability (Reinhart and Chien, 1986), the rhythmical discharge of ATP with the actual oxygenation status of hemoglobin might be a mechanism to augment muscular blood flow in horses if the transduction of shear stress alone is insufficient to enhance local blood flow during a race.

Camelid RBCs are elliptocytes (Eitan et al., 1976; Abdo et al., 1990; Moore, 2000) with major diameters in the range of 7–8 μm, and average minor diameters of 3.8–4.4 μm (Yamaguchi et al., 1987), depending on the camelid species. With the typical central depression of mammalian RBCs being absent, camelid RBCs have a constant thickness of 1.0–1.1 μm (Yamaguchi et al., 1987; Omorphos et al., 1989; Wernery et al., 1999). Their surface-to-volume ratio is only one third of human red cells (Khodadad and Weinstein, 1983) and their RBC volume is between 26 and 35 fL, depending on age (Wernery et al., 1999), which is less than in horses. The fatty acids of the membrane have shorter and more unsaturated hydrocarbon chains (Warda and Zeisig, 2001), which reduces membrane cohesion and facilitates membrane fluidity. The protein-to-lipid ratio of the RBC membrane is shifted toward the protein part (Eitan et al., 1976; McPherson et al., 1993; Al-Quarawi and Mousa, 2004), which increases the association of the bilayer with the cytoskeleton. The relative band 3 concentration per overall RBC surface area is threefold in camels compared to human (Khodadad and Weinstein, 1983), which has the potential to significantly stiffen the membrane. In camels, AE1 has a higher molecular weight (Ralston, 1975), a lower rotational and lateral mobility, and a tight connection to ankyrin (Khodadad and Weinstein, 1985; McPherson et al., 1993). As in horses, band 4.2 and band 6 appear to be absent (Omorphos et al., 1989). The elliptic shape and the smooth surface of camelid RBCs are maintained even at ATP depletion (Khodadad and Weinstein, 1983; Omorphos et al., 1989). However, the structures that maintain their shape during several stimuli that succeed in modifying the shape of round, biconcave RBCs are still unidentified. In principle, the network symmetry or functional aspects of cell deformability (Manno et al., 2005; Chu et al., 2016) must be different along the cell equator compared to its lateral faces on both sides, and structures must protect the membrane against detergents and permeation enhancers. A marginal band beneath the cytoskeleton on the cell poles or the equator might be present, but its occurrence in circulating cells has been controversial. Even when such a tubular system was detected (Abdo et al., 1990; Cohen et al., 1998), it was not found in all RBCs of an individual. In another study a marginal band was not detected at all (Goniakowska-Witalinska and Witalinski, 1976). Since the clinical picture of the tested animals with band bearing RBCs was inconspicuous, the cells were indeed either red cells, or they were reticulocytes in high numbers, but in contrast to human and other mammalian species this does not indicate enhanced erythropoiesis in camels.

The extrinsic cellular properties and the membrane composition mentioned above affect RBC stiffness and aggregability. By ectacytometry, elongation indices of horse RBCs are intermediate in a large series of mammalian species (Smith et al., 1979; Plasenzotti et al., 2004), and the shear stress that is needed for half-maximal red cell deformation belongs to the highest that have been measured so far (Windberger, 2019). Camel RBCs do not elongate at all in this test setup. This indicates that both mammalian cells are stiff, but only equine RBCs appear to elongate. The species difference is more pronounced in RBC aggregability. Horse whole blood can quickly build RBC clusters at low flow. The aggregation force between two neighboring cells is so high that even spiculated cells can be incorporated into a rouleaux formation (Baskurt et al., 1997). By contrast, the elliptic camel RBCs do not aggregate at all, which results in a low blood viscosity at low shear rates (Auer et al., 2015). The resting shape, the ability to withstand mechanical and osmotic stress, and intrinsic red cell properties reflect the diverse mechanical properties of camel and horse RBCs. A multitude of intact ghosts was found when a drop of native unfixed camel blood was completely dehydrated. Horse and human RBCs were completely lost under the same condition. Some of them became ghosts during indentation alone, an effect that has never been observed in camel blood.

Shape, membrane stiffness, and coupling to plasma significantly affect the red cell behavior in the vasculature and the resulting blood viscosity there (Abkarian and Viallat, 2016). Although an increase of hematocrit can enhance the dynamic organization of RBCs in macrovessels, an increase of oxygen carriers in individuals may not always be beneficial due to the effect of hematocrit on viscosity, especially if it is not balanced by a concomitant rise in plasma volume (El-Sayed et al., 2005). If a supplementation of erythrocytes is associated with a steep increase of viscosity, the hematocrit might soon be beyond an optimal value for the tissue that is in need of oxygen (Schuler et al., 2010). Since the flow behavior of red cells determines suspension viscosity (Abkarian and Viallat, 2016) we aimed to have a closer look at what can be expected in species with contrasting RBC properties, since camels and horses respond differently to strenuous exercise.

## Materials and Methods

### Animals

Ten dromedary camels (all female, age: 7–12 years) and 11 horses (5 mares, 5 stallions, 1 gelding, age 8–20 years, 1 Warmblood, 5 Thoroughbreds, 5 Arabians) were used. The dromedary camels were kept in large sandy paddocks in Dubai (United Arab Emirates). The horses were stabled in the surrounding area of Vienna (Austria) with daily access to a large pasture. All groups of animals had *ad libitum* access to food and water and were not subjected to specific training regimens, but were active without restriction in their paddocks. Blood was withdrawn from the camels in winter (January–February), and from the horses in spring (March–June). Ambient temperature ranged from 15–30°C during the time of the study. In order to observe the RBC behavior by microfluidics additional blood was withdrawn from three bactrian camels of an Austrian herd. The Austrian Federal Ministry of Science and Research (BMWF-66.009/0372-WF/V/3b/2014) approved the procedure on the horses and the bactrian camels. Blood sampling of the dromedary camels occurred during routine health checks of the herd.

### Blood Samples

Blood was withdrawn from the jugulary vein using a 21-gauge butterfly needle and a Vacuette blood collection system (Greiner Bio-One GmbH, Kremsmünster, Austria), containing K_2_-EDTA for anticoagulation. Hemograms were performed using Advia 2120i (Siemens Healthcare, Germany: horses) and Sysmex XT-2000i (Sysmex, Germany: dromedary camels) analyzers. For rheometry, blood was carefully centrifuged to separate plasma from RBC concentrate. Portions of blood plasma as well as concentrated RBC samples with varying hematocrit were immediately tested. The remaining amounts were mixed again to generate new samples of defined PCV (Hettich hematocrit centrifuge, Germany): 30, 40, 50, 60, and 70%. These samples [blood plasma, PCV adjusted samples, RBC concentrate (in five cases)] were tested in two steps. First, flow curves in simple shear were obtained at the following temperatures (*T*): 42 (camel only), 37, 32, 27, 22, 17, and 12°C, starting with the highest temperature. Second, a new portion of the sample was filled into the gap and sinusoidal shear was applied at the following temperatures: 37, 22, and 7°C, also starting with the highest temperature. All measurements were finished within a maximum of 10 h following blood withdrawal. Samples were stored at 7°C prior to their usage.

### Measurements

A Physica MCR302 rheometer (Anton Paar, Austria) equipped with a Peltier controlled stainless steel double gap cylinder system (internal gap: 0.417 mm; external gap: 0.462 mm, cup length: 42 mm) was used. The Rheocompass^*TM*^ software (version 1.19.335, Anton Paar, Austria) was used for data acquisition. In addition to rheometry, native blood samples were tested for RBC aggregation (aggregation indices M0, M1; Myrenne, Germany) at ambient temperature.

#### Simple Shear Flow Tests

Isothermal strain controlled flow curves (1000–10 s^–1^) were created by starting at the highest $\overset{\cdot }{\gamma }$ to obtain η of whole blood (WBV). The sample remained in the measuring system until completion of the last flow curve at the lowest temperature. To remove any pre-existing shear history, a 30 s pre-shear interval at 300 s^–1^ was set after each temperature equilibration. We started with the first flow curve at the highest temperature because it is closer to the physiologic body temperature of the animals (38.5°C in horse, between 35 and 41°C in camel). Shear viscosity was calculated from the stress-strain-dependency: η = τ/$\overset{\cdot }{\gamma }$ (Mezger, 2010).

Prior to this study a separate test was performed to determine if the order of the temperatures at which the flow curves are measured affects the test outcome. Native horse and camel blood was used for a series of flow curve pairs starting at 7°C and finalizing the measurement at 37°C, as well as starting at 42°C and finalizing at 12°C, respectively. The 37°C flow curve was compared to identify differences in viscosity measurements obtained during increasing or decreasing the temperature. In camel blood both flow curves were congruent. This indicates that there is no difference in WBV, irrespective of the starting temperature point of the experimental setting. However, error bars became high at shear rates below 10 s^–1^, which we attributed to the low viscosity of camel blood approaching the instrument detection limit. In subsequent tests we therefore set the shear rate range to a smaller interval from 1000 to 10 s^–1^ only.

A different result was seen in horse blood. A sudden decrease of low shear WBV (at shear rates between 0.1 and 20 s^–1^) was observed at temperatures between 22 and 32°C, if the experiment started at the low temperature setting. This sudden decrease of viscosity was associated with a decrease of shear thinning. No sudden viscosity increase was observed at any temperature range, when the experiment was started at the high temperature setting. In fact, the logarithmic viscosity/shear rate relationships increased in parallel manner with the temperature drop. To analyze if this phenomenon had any effect on the flow curves at 37°C, a two-sided *t*-test was performed (IBM^®^ SPSS, version 24.0). WBV at shear rates below 21.5 indeed depended on the temperature, at which the measurements had been started (10 s^–1^: *p* = 0.04; 21.5 s^–1^: *p* = 0.05), whereas viscosity values beyond 46.4 s^–1^ were indifferent. To be able to compare with camel blood, the identical shear rate intervals as for horse blood (from 1000 to 10 s^–1^) were used, starting at the high temperature setting. However, one must be aware that the first two data points on the flow curve (at 10 and 21.5 s^–1^) could be biased by the test procedure. There were no differences in viscosity if flow curves were performed strain or stress controlled.

#### Sinusoidal Shear Flow Tests

Stress-controlled tests were performed to obtain the shear moduli G′ and G^″^. Isothermal amplitude sweep and frequency sweep tests started at the highest temperature as before. Oscillation was applied to all PCV adjusted samples at 37, 22, and 7°C. The dynamic shear modulus (G^*^) is calculated from the stress-strain-relationship during the sinusoidal change of time and amplitude (G^*^ = τ(ω,A)/$\overset{\cdot }{\gamma }$(ω,A)). The phase shift angle (δ) shows the lag phase between the applied stress and the resulting strain and indicates therefore the in-phase and out-of-phase components of G^*^. Multiplication of G^*^ with cos(δ) determines the G′, and multiplication with sin(δ) the G^″^. Subsequently G′ and G^″^ are stated (Mezger, 2010).

Amplitude sweep tests were set at 10 rad⋅s^–1^. After the pre-shear interval (30 s oscillation at 0.01 Pa and 10 rad⋅s^–1^) increasing shear stresses of 0.001–10 Pa were applied. The maximum shear stress that indicated the end of the linear viscoelastic range out of the G′-curve was defined as yield stress of our suspensions. Such yield points were estimated by using the Rheocompass^*TM*^ software or manually.

Following a further pre-shear interval (30 s oscillation at 0.01 Pa and 10 rad⋅s^–1^) frequency sweep tests were performed at 0.01 and 0.005 Pa, depending on the PCV, reflecting linear test mode for horse blood. As recently demonstrated (Windberger et al., 2017), only a few frequencies can be applied successfully for frequency sweep tests in linear mode on blood. Therefore, a rather narrow range of 2.0–0.5 Hz was used, starting with the highest frequency. For data analysis the shear moduli between 0.5 and 1.0 Hz were used (two cumulating data points), resembling the physiological heart rate of the animals. The obtained shear moduli were pooled for each PCV – temperature combination and used for species comparison.

#### Microfluidic Tests of Camel Blood Suspensions

Samples of 1% PCV were prepared in autologous plasma. RBCs flowed through straight channels of a polydimethylsiloxan-based microfluidic device drew from and flowed into a common reservoir with an approximate height of 1 mm. The microfluidic device contained several parallel channels with cross sectional dimensions of ∼12 × 10 μm in width and height, respectively, and a length of approximately 4.0 cm each. A pressure device (Elveflow, OB1, France) was connected to an external reservoir that contained the camel RBC sample. A polyethylene tube connected the external reservoir to the inlet of the microfluidic device from where the cells were injected. The sample was subjected to three different pressure drops of DP = 100, 1000, and 1500 mbar to induce different cell velocities. Cells were observed in an inverted microscope (TE2000, Nikon, Japan) using an air objective with 60-fold magnification and a numerical aperture of 1.4. With the help of a motorized xy stage, two different positions were observed along the channels: the entrance or position 0 and a second position 10 mm inward from the channels entrance. Images were recorded using a high-speed camera (Phantom Miro LC310, Vision Research, United States) at 100, 800, and 1000 frames per second for DP = 100, 1000, and 1500 mbar, respectively. A self made MATLAB routine was used for image processing and quantitatively data extraction. We define two cells to be a part of a cluster if the shorter distance among their surfaces was equal or less 8 mm (typical long axis length of a camel RBC), following the definition used in previous studies on human RBC cluster formation (Tomaiuolo et al., 2012).

### Statistical Evaluation

Sample size calculation was performed on data obtained from four horses (Peters, 2016). Viscosity values were used to simulate a sample size of 5, 10, and 15 by several bootstrap steps. The 95% confidence intervals were estimated for the available PCV- and temperature-combinations by using a linear mixed model. Thereafter, the distance from the confidence interval limits to the estimated mean values were defined as follows: 0.75 mPa⋅s at 1000 s^–1^ and 2.00 mPa⋅s at 10 s^–1^. The value at which the confidence interval half amplitude was smaller than this defined distance determined the sample size of *n* = 10. The same sample size determination method was used for both camels and horses.

To model the viscosity of the two species, data clearing by LibreOffice (version 6.1.2.1) (The Document Foundation, Berlin, Germany) was done prior to the regression analysis. A total of nearly 13000 values were obtained from camel and horse. Some values were deleted as described in the following. By presuming that blood viscosity must show a monotonical curve when the shear rate increases, such values that differed from the proceeding or the successive value by more than 50% were declared as a spike and deleted (711 values from camels and 396 values from horses). Viscosity values displayed by the Rheocompass software^*TM*^ associated with sudden changes in the normal force in the gap were excluded as well (23 values from camels, none from horses). Finally, mean values were calculated per condition (shear rate, *T*, PCV).

### Modeling of Viscosity

The power law relationship between shear rate and viscosity formed the basis of the modeling. Equation (1) depicts the resulting equation for camels; Eq. (2) shows the relationship for horses. PCV affected viscosity exponentially in both species. Whereas temperature affected viscosity by power law in camels, it influenced viscosity in the manner of a third degree polynomial function in horses. Two additional terms were included to Eq. (2) to fit the dependency on combinations of shear rate and temperature, as well as shear rate and PCV in horses.

(1) $$\eta =1.25+3.176\cdot {\overset{\cdot }{\gamma }}^{-0.047}\cdot T^{-0.841}\cdot e^{\left(0.074\cdot P\,C\,V\right)}$$

(2) $$\eta =4.331\cdot \left(6.292\cdot {\overset{\cdot }{\gamma }}^{-0.65}-0.022\cdot \log\left(\overset{\cdot }{\gamma }\right)+1\right)\;\cdot \left(0.00002101\cdot T^{3}+0.00176\cdot T^{2}-0.0592\cdot T+1\right)\;\cdot e^{\left(0.0373\cdot P\,C\,V\right)}\cdot e^{\left(\frac{1}{{\overset{\cdot }{\gamma }}^{\left(0.02\,T+0.46\right)}}+\frac{1}{{\overset{\cdot }{\gamma }}^{\left(0.662\cdot 10^{12}\cdot P\,C\,V^{-6.44}\right)}}\right)}$$

The coefficients were calculated by non-linear least-square regression (Strutz, 2015) that was applied to the mean values after data clearing. Statistic and modeling was performed by Gnu R (version 3.5.1) (R Core Team, 2018). Figures were performed by Gnu Plot (version 5.2.4) (Williams et al., 2018). Modeling is only valid within the ranges that we used for the measurements: camel: PCV (30–60%), *T* (12–42°C), shear rate 10–1000 s^–1^; horse: PCV (30–70%), *T* (12–37°C), and shear rate 10–1000 s^–1^. It should be noted that the formulas are not phenomenological expressions, but describe the experimental data.

### Limitations of the Study

Since we used whole blood from adult animals of both species, the obtained results reflect this age group only. Additionally, the dromedary camels lived in their indigenous desert habitat at sea level and specimens were randomly selected from a herd of about 300 animals. Different habitats and narrow gene pools have the potential to modify both geno- and phenotype; therefore rheometric properties of a small herd of dromedary camels housed in a mild climate might be different. If our observations are anything to go by, the dromedary camels′ breed itself is not likely to influence the outcome, since four random samples of another dromedary camel breed (Pak camels with dark brown fur), kept in a Dubai dairy farm, showed identical results. Finally, our results cannot be considered generally for all camelid species, even though all members of this family have elliptic RBCs.

## Results

### Tests in Simple Shear Flow

**FIGURE 1:**
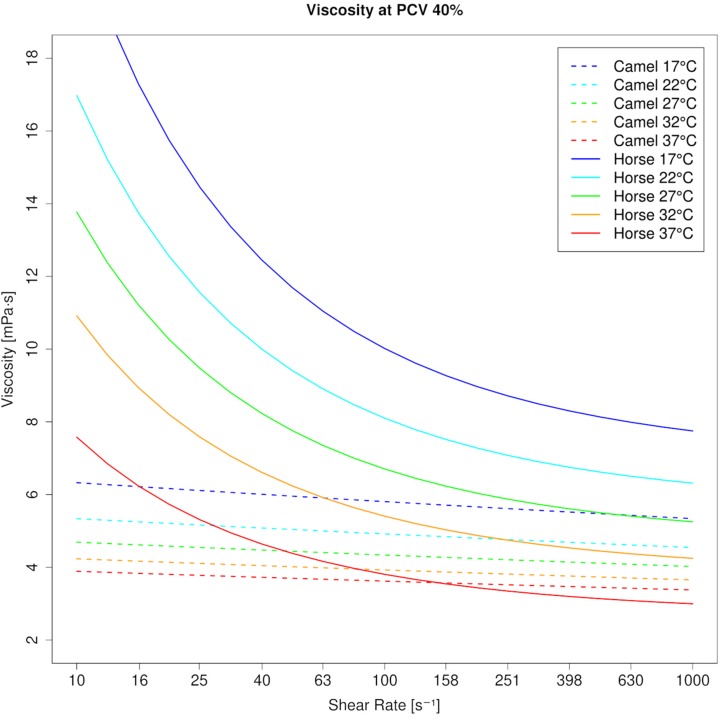
Viscosity curves of camel blood and horse blood at 40% PCV (modeled values). Whereas camel blood is almost Newtonian, horse blood shows shear thinning. Temperature has a greater effect on horse blood viscosity than on camel blood viscosity at each shear rate.

**FIGURE 2:**
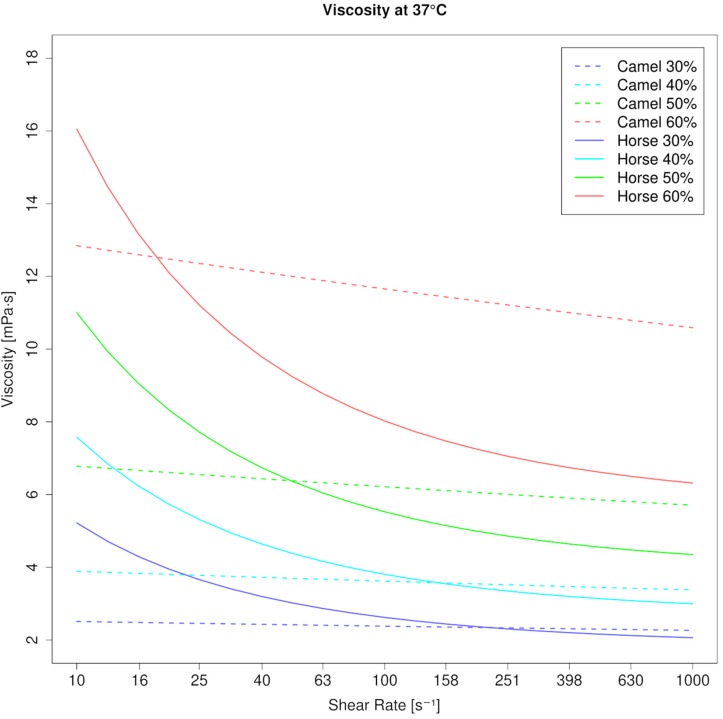
Viscosity curves of camel blood and horse blood at 37°C (modeled values). Increase of viscosity in camel and horse blood suspensions with increments of PCV. At native PCV (30% and beyond) camel blood shows lower viscosity than horse blood. But when the PCV rises, viscosity becomes higher in camels. Note the steep increase of viscosity at the high shear rate (1000 s^–1^) in camel blood suspensions. Such an increase of viscosity along with the PCV is avoided in horse blood through its shear thinning property.

**FIGURE 3:**
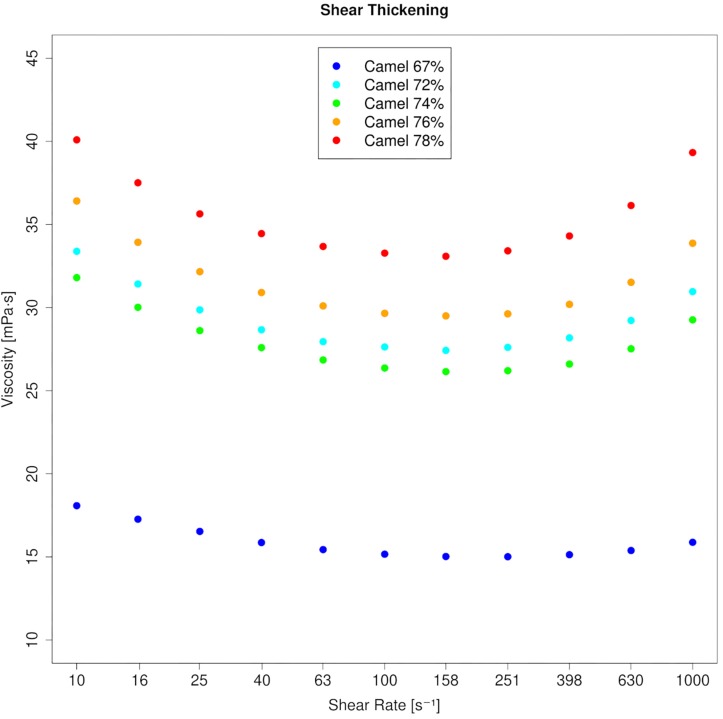
Viscosity curves of RBC concentrates from five dromedary camels. Profound shear thickening occurs at PCVs beyond 70%. A linear *y*-axis was chosen to better illustrate the effect.

Blood viscosity (WBV) in camels was 3.74 mPa⋅s at 10 s^–1^ and 3.44 mPa⋅s at 1000 s^–1^ (40% PCV, 37°C), which indicates camel whole blood as a very weak pseudoplastic fluid. This is also shown by the low negative coefficient for shear rate (γ = −0.047). In contrast, horse blood showed the typical shear thinning behavior with WBV being 7.22 mPa⋅s at 10 s^–1^ and 2.99 mPa⋅s at 1000 s^–1^ at equal condition (40% PCV, 37°C; coefficient: −0.65). The effect of temperature on blood viscosity was pronounced in the horse regardless of the shear rate (Figure 1). However, the effect of PCV on blood viscosity depended on shear rate and species. At low shear rate, horse blood viscosity was generally more prone to changes in PCV. But at high shear rate, the increase of blood viscosity with the PCV was augmented in the camel (Figure 2). In the RBC concentrates, shear thickening was present in camel blood samples (see Figure 3), but not in horse blood samples. The hemogram, the whole blood and PV values, and the aggregation indices of the RBCs of the two species are depicted in Table 1.

**TABLE 1:** Red blood cell indices (MCV, in fL; MCH, in pg; MCHC, in mg dL^–1^) and aggregation indices (M0, M1) were obtained from the native blood samples, and the samples were measured at ambient temperature. Shear viscosity (η), shear moduli (G′, G^″^, obtained in linear mode), and yield stress of whole blood suspensions are shown at three PCV-values. The displayed data were measured at 37°C.

**⋅**	**Camel (*Camelus dromedarius*)**		**Horse (*Equus caballus*)**	
**RBC indices, plasma viscosity, and RBC aggregation indices**				
MCV	30 (28/31)		45 (43/46)	
MCH	14.3 (13.5/14.5)		16.6 (15.9/16.9)	
MCHC	47.7 (46.3/48.2)		38.8 (38.0/39.3)	
PV	1.04 (1.00/1.07)		1.62 (1.58/1.67)	
M0	0 (0/0)		13.2 (11.8/14.4)	
M1	0.55 (0.07/1.57)		56.9 (44.2/68.3)	
**Shear viscosity of whole blood suspensions in simple shear flow (in mPa s)**				
	η at 10 s^−1^	η at 1000 s^−1^	η at 10 s^−1^	η at 1000 s^−1^
PCV 40%	3.74 (3.65/3.96)	3.44 (3.39/3.61)	7.22 (6.89/7.95)	2.99 (2.83/3.18)
PCV 50%	6.68 (6.48/6.82)	5.90 (5.79/6.03)	11.51 (10.85/12.89)	4.18 (3.92/4.49)
PCV 60%	12.81 (12.47/12.97)	10.81 (10.65/11.22)	18.50 (16.43/19.87)	6.16 (5.61/6.62)
**Shear moduli of whole blood suspensions in sinusoidal shear flow (in mPa)**				
	G′ at 1 Hz	G^″^ at 1 Hz	G′ at 1 Hz	G^″^ at 1 Hz
PCV 40%	NA	27.1 (26.3/28.5)	20.2 (16.7/28.6)	59.1 (54.6/62.3)
PCV 50%	NA	35.7 (35.5/39.1)	44.3 (40.8/44.3)	96.9 (93.2/97.5)
PCV 60%	NA	58.5 (57.8/61.3)	78.1 (70.8/87.5)	140.0 (135/145)
**Yield stress of whole blood suspensions (in mPa)**				
PCV 40%	NA		7.175 (4.58/9.925)	
PCV 50%	NA		9.81 (4.56/9.92)	
PCV 60%	NA		33.79 (18.58/46.16)	

### Tests in Sinusoidal Shear Flow

#### Horse

G′- and G^″^-values of horse blood were increasing functions of frequency at each PCV and temperature setting. Horse blood remained a viscoelastic liquid even at 70% PCV. Percolation occurred in the RBC concentrates only. Loss factor values (G^″^/G′) between 0.5 and 1.0 Hz of nine concentrated horse blood samples were 1.07–0.67, where the 79% PCV sample showed the higher and the 85% PCV sample the lower tanδ values. At 10 rad s^–1^, horse blood yielded. All elasticity was lost beyond 0.2 Pa. G′, G^″^, and yield stress increased with PCV.

#### Camel

**FIGURE 4:**
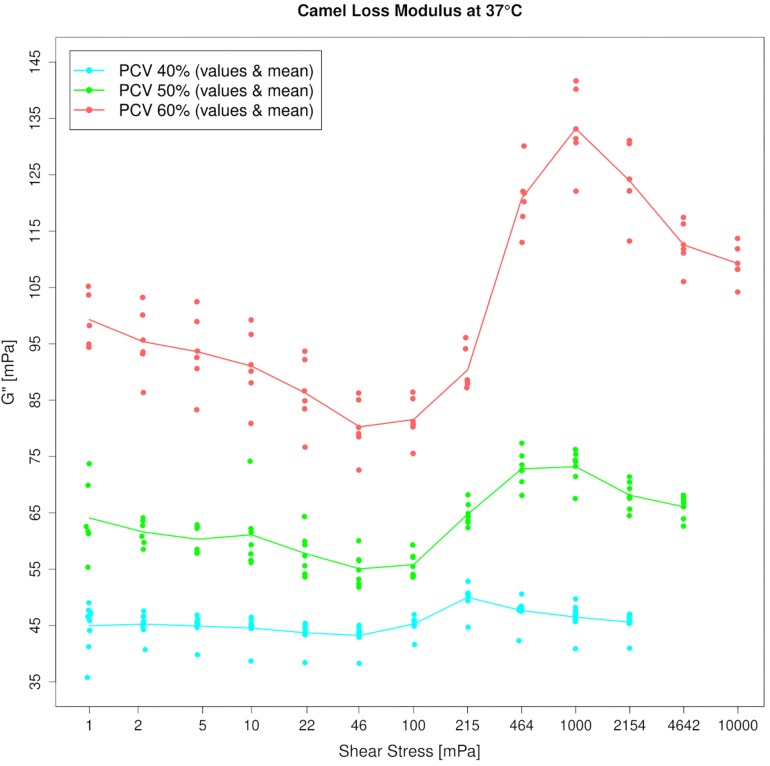
Amplitude sweep test of camel blood suspensions at 1 Hz. Loss moduli (G^″^) increase beyond 50 mPa which indicates energy dissipation. The effect is enhanced by addition of red cells. Please note the linear *y*-axis.

In contrast, G′-values could not be measured in camel blood because the phase shift angle during the measurement was too close to 90°. A G′-value was absent even when the PCV was increased to 60%. To generate a lower phase shift angle that would allow displaying elasticity in the sample, PCV had to be increased beyond 75%, or temperature had to be lowered to 7°C. Only at such drastic parameter settings most (but still not all) camel blood samples exhibited G′ in our test system. Since these conditions are far away from being physiologic, it indicates that flowing camel blood does not yield in the body. Only if RBCs would be locally concentrated to very high concentrations, camel blood might display a yield stress. G^″^ was an increasing function of frequency like in the horse and increased with the hematocrit. In contrast to horse, at amplitudes beyond 1 Pa (Figure 4) a local G^″^ maximum was present in the camel blood samples that increased with the PCV.

Table 1 presents the G${}_{1}^{\prime }$_*Hz*_ and G${}_{1}^{\prime \prime }$_*Hz*_ values and the yield stresses of the suspensions.

**FIGURE 5:**
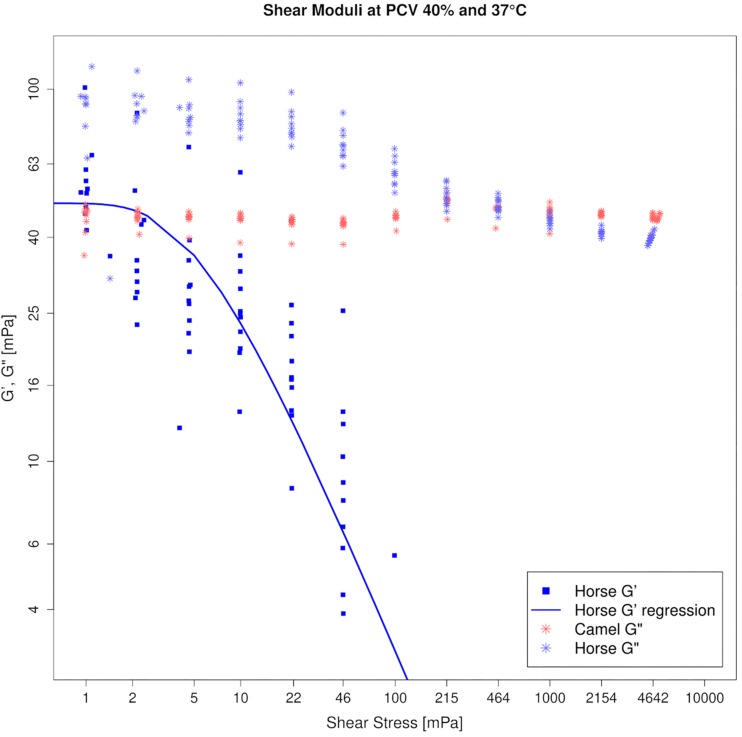
Amplitude sweep test of horse (blue) and camel (red) blood suspensions at 40% PCV on log–log axes, tested at 37°C. Storage modulus (G′) is absent in camel, revealing that camel blood is purely viscous at this condition. Therefore only G^″^ can be shown for the camel in this figure. Horse blood is a viscoelastic threshold fluid, displaying a yield stress given in Table 1.

Figure 5 depicts the amplitude sweep tests for horse and camel whole blood at 40% PCV and 37°C.

### Microfluidic Tests

**FIGURE 6:**
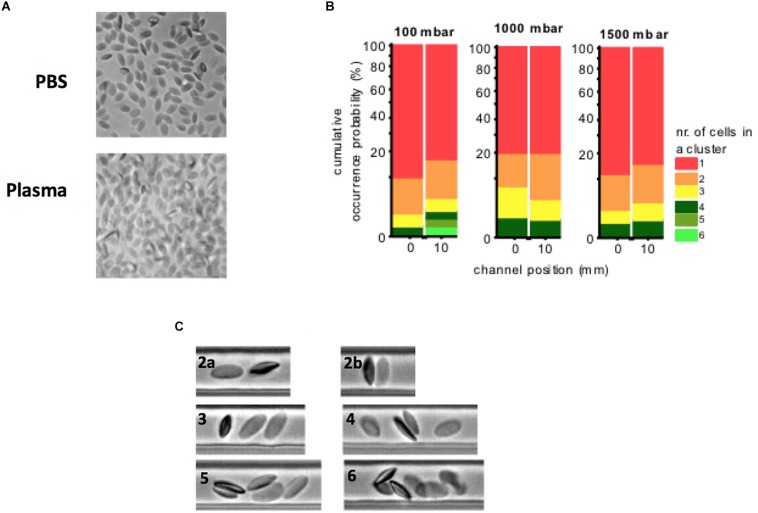
**(A)** Camel RBCs at rest suspended in their autologous plasma and buffer solution (PBS). No aggregates are observed. Several RBCs preferentially adhered with their thinner lateral face to the glass slip. **(B)** Camel blood suspensions (1% PCV in their plasma): no cluster formation was observed at 1000 and 1500 mbar, a slight cluster formation was observed at 100 mbar, resulting from a higher residence time that would allow RBCs to encounter sporadically. **(C)** Cluster examples (2a–6): only transient clusters were observed in each case. Clusters were not stable in time.

No cluster formation was observed at 1000 and 1500 mbar, only a slight cluster formation was observed at 100 mbar, resulting from a higher residence time that would allow RBCs to encounter sporadically (Figure 6). Only occasionally transient clusters were observed that were not stable over time (Supplementary Video S1). Table 2 shows the camel’s mean RBC velocity in the straight channel at a feeding PCV of 1%.

**TABLE 2:** Camel’s mean RBC velocity (with the standard deviation indicated) at a feeding PCV of 1% in autologous plasma at three different pressure drops (DP).

**Camel’s RBC velocity (in mm/s)**				
	**Channel position (in mm)**			
**DP (in mbar)**	**0 (Entrance)**		**10**	
	**Mean**	**SD**	**Mean**	**SD**
100	0.64	0.07	0.6	0.07
1000	4.82	0.6	6.69	0.83
1500	5.86	0.74	7.72	0.98

## Discussion

A weak pseudoplastic (in fact almost Newtonian) behavior of blood suspensions, as evidenced in this study, is unique among the mammalian class. Keeping in mind that the blood samples contacted rigid steel surfaces in our standard rheometrical test setup, which modifies the effects of the physiological containment on the circulating blood volume like RBC migration and deformation (Kumar et al., 2014), a species-specific shear thinning was always present in other mammalian blood samples that have been tested so far (Windberger, 2019). Increasing PCV in dromedary in camel blood did only marginally modify this characteristic (see Figure 2). It appears as if cell crowding does not raise the effective shear rate between the cells enough to force their orientation and to result in a more collective dynamic motion of the cells. The forces that are usually transmitted from one cell to the other by the displaced fluid might also be too unstable due to RBC tumbling, even if the PCV is high. However, camel blood always remained a viscous liquid without a threshold unless very high volume fractions were tested, at which a sudden transition occurred. The extreme non-physiological conditions that were necessary to generate elasticity in the samples included high volume fractions (>75% PCV) in combination with fridge temperatures. These findings were in clear contrast to those found in horse blood. Horse blood suspensions showed a threshold (see Figure 5), which typically increased with the PCV (Windberger et al., 2017). It is obvious to associate such yielding with RBC aggregation (Lee et al., 2007), however, this cannot be the only cause. Even higher yield stresses than those found in horse blood were found in pig and rat blood suspensions adjusted to 40% PCV (Windberger, 2019). Both species show inferior RBC aggregability compared to horses (Windberger et al., 2003). According to the DLVO theory, yield stress is a balance between entropic and kinetic forces (Verwey and Overbeek, 1948). Short distances between particles favor van der Waals attraction, whereas components of the double layer generate their repulsion (Hunter and Nicol, 1968; Russel et al., 1989). High surface charges of RBCs will therefore increase the distance between neighboring cells and weaken the cell–cell attraction. A cell’s surface charge can be estimated by the amount and concentration of sialic acids stemming from the carbohydrate rich proteins in the membrane. For both species the RBC sialic acid content is provided (Khodadad and Weinstein, 1983; Bäumler et al., 2001), but due to the different techniques used, the given values are not comparable. In both papers, however, the amount of sialic acid was higher in camelid and horse RBC membranes than in human red cell membranes. A kind of “effective” surface charge was estimated by using a method to distribute RBCs between two liquid phases of different hydrophilicity. Equine RBCs accumulated in the phase with lower hydrophilicity, and the authors postulated from this finding that the greater aggregation tendency of horse RBC could be associated with a lower surface charge (Baskurt et al., 1997). This might explain at least in part the differences in yielding between camel and horse bloods since the cell–cell repulsion affects the colloidal microstructure at those shear forces where yielding occurs. Preliminary studies in straight microfluidic channels showed that the camel RBCs formed clusters only by chance when they narrowed each other. When such a cell assembly was transported through the channel the clusters separated again (see Supplementary Video S1). This explains the lack of yielding in this species.

The search for the underlying reason of this particular bulk behavior of camel blood may start at the mechanical properties of the red cells. Since elliptic RBCs do not show the typical increase of elongation indices at increasing shear stresses by ectacytometry like biconcave cells (Smith et al., 1979) that would allow a comparison of maximum cell elongation, single cell techniques are needed to measure deformability of camelid RBCs. By single cell force spectroscopy, camel RBCs showed a higher median value of apparent Young’s modulus than horse RBCs. Horse RBCs in turn showed a significant unexpected stiffness when compared to human red cells (Baier et al., 2016). This is surprising given the high aggregability of equine red cells, but already known from ectacytometry (Plasenzotti et al., 2004). In camels, high red cell stiffness was expected from the fortification of the membrane by the amount and the stability of the vertical linkages between the bilayer and the cytoskeleton (Khodadad and Weinstein, 1983). It is well known that rigid particles subjected to simple flow rotate around their axis with the angular velocity of the suspension medium (Bretherton, 1962). Such tumbling occurs in tubes that are significantly larger than the cell size and was also described for rigid RBCs (Goldsmith and Marlow, 1972). Tumbling occurs frequently during creep, even if RBCs are highly flexible. But when the shear rate increases, more coordinated motions are feasible for the cell, which allow its alignment in the flow field. The full phase diagram of RBC motion and orientation by considering the influence of capillary number and viscosity contrast is provided for biconcave RBCs (Abkarian and Viallat, 2016). For instance, the stress or strain threshold at which a rigid RBC starts to switch from tumbling into rolling depends on the relationship between the elastic energy of the membrane and the ability of the bilayer and the cytoplasm to dissipate viscous forces that are applied to the cell by the surrounding fluid flow (Abkarian et al., 2007; Skotheim and Secomb, 2007; Forsyth et al., 2010; Fedosov et al., 2014). The cytoskeleton and its anchors to the bilayer provide this elasticity, whereas the membrane and cytoplasm fluidity are responsible for the uptake of viscous forces (Lim et al., 2008). A cell whose membrane is not fluid enough to displace its elements will not be able to keep a fixed angle relative to the direction of the streamlines in a vessel (Dupire et al., 2012; Wirther et al., 2012). Due to the constant change in RBC orientation the suspension will thus generate a higher viscosity. This is exactly what was observed beyond a certain shear rate in the camel blood.

If the cell shape relative to the direction of the flow cannot be maintained, a flat elliptic cell that tumbles will occupy a larger space compared to a round cell of the same volume. It would collide with neighboring cells more frequently if the packing fraction is high enough. And if coupling does not occur, the effect will rise with the blood flow velocity. This could explain the strain overshoot in the loss modulus (between 0.05 and 1 Pa) that indicates energy dissipation, which was present even at low PCV, but could be augmented with the addition of RBCs (Figure 4). The shear thickening behavior seen in simple shear flow, which is also correlated to energy dissipation processes, was only seen at very high volume fractions. Shear thickening is not a common finding in blood suspensions. In fact, it can be achieved even in elliptic, nucleated RBCs only after aldehyde hardening (Usami et al., 1970). In hard-sphere colloids, shear thickening occurs when particles aggregate into closely connected clusters that are dynamically trapped by lubrication forces (Wagner and Brady, 2009; Israelachvili, 2011). Therefore, its occurrence in camel blood indicates a transition toward a clustered microstructure at higher flow when a PCV threshold is passed. But a sudden jamming also occurred at low stress after the same PCV threshold. There was no elasticity in the camel blood samples at quasi-static conditions as long as the PCV was equal or below 60%. But in all RBC concentrates with PCVs of 70% and above, a storage modulus appeared with values not much different from horse blood samples at such high volume fractions. A distinct difference between the species was evident in the progression of G′ which in the horse blood samples occurred concomitant along with the PCV increase, whereas it emerged suddenly in the camel blood samples. Therefore, a sharp transition of the microstructure of the suspension must occur between 60 and 70% in both, low and high flows in camel blood (while structures might evolve continuously in horse blood), although its exact nature cannot be determined at present. Hypothetically, the RBCs stack with a certain offset and form blocks with varying microscopic anisotropy when exposed to a stress modulus. Apart from the importance to the basic physiology of dromedary camels, this finding makes their blood suspensions a valuable model for investigating the behavior of elliptic colloids in flow.

Another factor that has to be considered in this context is the viscosity contrast across the red cell membrane, because it determines the coupling between cells and plasma in flow. Camel PV was only marginally higher than the viscosity of water and the lowest among a series of many mammals that were tested (Windberger et al., 2003); but the MCHC of camel RBCs was surprisingly high. This generates a high viscosity contrast and is another factor in addition to membrane stiffness that affects the response of the cells to shear flow (Fedosov et al., 2010; Zhang, 2011). When RBCs flow in a cylindrical tube, the surrounding fluid exerts a tangential stress on the RBC membrane. This force scales with the hydraulic pressure and the PV, and in human RBC membranes it generates tank treading. Tank treading allows the cell to keep its biconcave shape while it is flowing before it starts to deform at higher stresses, and aids in maintaining its orientation angle. A viscosity contrast below a certain limit therefore favors the coupling between fluid flow and RBC membrane, and via augmentation of RBC membrane dynamics viscosity can be lowered (Fedosov et al., 2014). The importance of PV has been well documented. Tran-Son-Tay et al. (1984) observed that the tank treading frequency increased at higher PV. Fischer et al. (1978) demonstrated that RBCs suspended in a low viscosity medium tumble, but they start to tank tread if the viscosity of the medium is sufficiently high. Keller and Skalak (1982) analyzed the motion of a viscous ellipsoid and concluded that the behavior depended on the ratio of viscosities of the inner and outer fluids and was nearly independent on the shear rate. Later, Fischer and Korzeniewski (2011) showed that the PV played even a greater role than the shear force to elongate RBCs. The high viscosity contrast across the camel RBC membrane and the rheological behavior of the bulk (the low shear thinning) implicate that membrane motions will be low compared to other mammals. This must originate at least in part from the tight binding of the band 3 proteins to the underlying ankyrin residues, Since the cohesion between the phospholipids will be low (Warda and Zeisig, 2001), the bilayer will not hinder the shift of proteins. Low membrane motions promote uncoordinated cell motions in shear flow and explain our rheological findings.

But how can camel RBCs with such seemingly impossible properties flow through the microvasculature? Once having entered into capillaries, the cells should flow easily through them due to their elliptic shape resembling an already elongated cell. But from the aforementioned it is apparent that crowding of RBCs at the entrance to small vessels must be avoided. This can be achieved by a low feeding hematocrit in front of the bifurcation or a twisted vessel. It was observed that the elliptic red cells of Peking ducks were excluded from flowing through narrow glass capillaries by unsorted cell crowding in front of the inlet (Gaehtgens et al., 1981). Camel RBCs are stiffer than other mammalian cells (Waugh, 1992) and obviously they do not align in streamlines. How they arrive at bifurcations cannot be predicted. Extrapolating from observations in microfluidic devices (Zhang et al., 2019), the bending rigidity of camel RBCs might be causative for capillary entrance. Cell bending is a non-linear response to strong deformations mediated by a change in symmetry of the spectrin cytoskeleton (Gov et al., 2003). In camelid RBCs the connections of the cytoskeleton to the membrane are tight (Khodadad and Weinstein, 1983), which implies significant bending rigidity and difficulties to flow into kinked geometries. However, there is no data available to confirm this hypothesis. Since the very weak shear thinning implicates that camel RBCs flow rather as separate entities, a collective behavior cannot be expected, and crowding might occur in front of obstacles. These causal relationships could explain the low physiological hematocrit in camels.

A problem that arises in racing camels is the occurrence of anemia associated with fluctuations of plasma iron concentrations, which are treated by the veterinarians with iron supplementations and in some instances also with homologous blood transfusions (Cluer et al., 1994; Rose et al., 1994; Tinson, 2017). The question arises if the anemia in racing camels could be a mechanism to relieve the microvasculature from effects that can be generated by a high RBC count. At high shear rates – e.g., at high cardiac output during a race – there is a pronounced rise in blood viscosity along with the PCV. Any increment of hematocrit raises the cardiac pressure-volume workload in the camel more effectively than in other mammalian species. Although increasing the amount of circulating RBCs immediately increases the oxygen transport capacity, enhancing performance of athletes, it is unclear what the long term costs are based on the remodeling of the cardiovascular system (Cecci et al., 2011).

On the other hand, the weak pseudoplastic behavior also offers advantages. Native camel blood (PCV was lower than 30% in most of the camels studied) shows a lower viscosity at all shear rates when compared to horses. The results show that camel WBV is only half of that of horses at low strain, due to camel RBCs not forming aggregates. The low PV contributes further to the low WBV. In this model, camel blood did not yield under any experimental setting. Applying this observation to the real vascularity indicates that blood is always viscous, even when the flow approaches zero. Blood in venules and other vascular geometries with large diameter could become sluggish by a rise in PCV. But in camels this property is maintained even if the hematocrit rises to extreme values. The ability of camel blood to maintain viscous even at creeping flow can be a benefit for the animals since it could support lactate washout even in phases of rest. Such a mechanism could be the result of the remarkable adaption of this species to the harsh natural environment. However, to maintain blood fluidity seems to be very important for camels. A long-term water-deprived camel living in the Sahara desert lost even more than 20% of its body weight while the animal could maintain its circulating blood volume. A dye dilution technique revealed that dromedary camels loose water from tissues but not from the circulating blood (Schmidt-Nielsen, 1959). As species can modify their phenotype in response to the environment it cannot be predicted if animals transferred to mild climates show this feature as well, since several physiological, life-saving mechanisms in dromedary camels are responses to harsh environments (Gaughan, 2011). Our data show typical features of adult dromedaries living in their natural habitat.

In summary, whole camel blood shows low viscosity at physiological hematocrit values and an absence of yielding even at high RBC count, indicating low cohesion between its components, which is reflected by the near-Newtonian behavior of the blood suspensions. The high viscosity contrast across the RBC membrane points to a weak coupling between flow and cell behavior and can increase the inclination angle of the flowing RBCs, which increases blood viscosity. Subjected to simple shear, we predict that camel RBCs flow as separate entities that might even show various types of motions. This can lead to friction instead of a collective alignment to the direction of the flow. This behavior will become clinically relevant at higher RBC count, since the high shear viscosity rises through a much greater coefficient with the hematocrit compared to horses. At high shear conditions in the vasculature, any infusion of RBCs into the bloodstream therefore has the potential to increase the cardiac work and the energy input into the endothelium more effectively in camels compared to other mammals. The effect of racing on the incidence and severity of pulmonary hemorrhage is one issue that cannot be neglected (Akbar et al., 1994). Although the performance of athletes can be enhanced by the addition of circulating RBCs, a specific RBC phenotype is required to tolerate it at long term. And since a rise in PCV can occur in parallel with the running speed of camels (Evans et al., 1994), our study might encourage camel trainers to maintain the hematocrit in their animals below 35%. The relevance of these findings in regard to the muscle fatigue of working and racing camels will be subject of further studies.

## Data Availability

The datasets generated for this study are available on request to the corresponding author.

## Ethics Statement

The Austrian Federal Ministry of Science and Research (BMWF-66.009/0372-WF/V/3b/2014) approved the procedure on the horses and bactrian camels. Blood sampling of the dromedary camels occurred during routine health checks of the herd.

## Author Contributions

UW planned the study, designed the experiments, and performed the data acquisition and analysis on camel blood. RA performed the data acquisition and interpretation on camel blood, and edited the manuscript. MS performed the data acquisition and analysis on horse blood. GM modeled the viscosity and analyzed the data. JS contributed to the conception of the work and revision of the manuscript.

## Conflict of Interest Statement

The authors declare that the research was conducted in the absence of any commercial or financial relationships that could be construed as a potential conflict of interest.

## Supplementary Material

VIDEO S1 Clusters are transient and not stable in time. The flow was produced applying a pressure drop of 1000 mbar. The mean cell velocity was 6.99 ± 0.83 mm/s.

## Abbreviations

$\overset{\cdot }{\gamma }$: shear rate
η: shear viscosity
DP: pressure drop
G ′: storage modulus
G ^″^: loss modulus
MCH: mean cellular hemoglobin
MCHC: mean cellular hemoglobin concentration
MCV: mean cellular volume
PCV: packed cell volume
PV: plasma viscosity
RBC: red blood cell
*T*: temperature
WBV: whole blood viscosity.
